# Morphological Shift and Lipid Accumulation in *Trichosporon cutaneum* B3 Induced by Enhanced Dissolved Oxygen

**DOI:** 10.3390/jof12050312

**Published:** 2026-04-24

**Authors:** Ya Wang, Bin He, Riming Yan

**Affiliations:** 1 College of Life Sciences, Jiangxi Science and Technology Normal University, Nanchang 330013, China; 2 Key Laboratory of Biodiversity Conservation and Bioresource Utilization of Jiangxi Province, College of Life Sciences, Jiangxi Normal University, Nanchang 330022, China

**Keywords:** *Trichosporon cutaneum*, dissolved oxygen, microporous distributor, microbial oils

## Abstract

In oleaginous yeast submerged fermentation, dissolved oxygen (DO) regulates both metabolism and cell morphology. Under oxygen limitation, *Trichosporon cutaneum* transitions from yeast-form to hyphae-form; the yeast-form morphology is more suitable for lipid production. This study enhanced oxygen transfer via reactor engineering to maintain yeast morphology and improve lipid productivity. Three strategies were assessed: increased agitation/aeration, enriched air supply, and microporous ceramic membrane gas distributor (MCMGD). Fermentation kinetics were analyzed alongside computational fluid dynamics (CFD) simulations of volumetric mass transfer coefficient (*k*_L_a), gas holdup, bubble diameter, and flow fields. Conventional strategies only partially alleviated oxygen limitation (maximum 4.47 g/L lipid). Enriched air improved lipid content but induced early myceliation. The MCMGD (1.0 vvm, 150 rpm) shortened fermentation from 150 h to 60 h, achieving 12.06 g/L lipid (49.16% content)—a 2.16-fold lipid concentration increase. Mechanistically, it generated smaller bubbles (1.47 mm vs. 2.54 mm) and higher *k*_L_a (0.012 s^−1^ vs. 0.0055 s^−1^). CFD revealed improved axial flow, reduced dead zones, and uniform gas holdup, suppressing yeast-to-hyphae shift. By enhancing mass transfer under low shear, the MCMGD ensures adequate oxygenation, maintains productive morphology, and significantly improves lipid production—offering a promising strategy for industrial application.

## 1. Introduction

The excessive extraction and use of fossil fuels present two major challenges: growing concerns over resource depletion and the increasingly severe issue of climate change caused by greenhouse gas emissions [1,2]. Against this backdrop, biofuels produced from renewable biomass resources—such as bioethanol and biodiesel—have garnered significant attention as promising alternatives [3,4]. Biodiesel, in particular, can be produced by microorganisms converting inexpensive feedstocks like agricultural residues and lignocellulosic biomass. This carbon-neutral pathway offers unique advantages in terms of sustainable resource utilization and avoiding competition with food crops [5,6].

Through oil extraction and transesterification, microorganisms can convert the triglycerides they produce into fatty acid methyl esters, which are the primary components of biodiesel [7]. Among microorganisms suitable for biodiesel production, oleaginous yeast has emerged as a research hotspot due to its rapid growth and high lipid accumulation efficiency [8]. *Trichosporon cutaneum* stands out as a highly promising candidate, characterized by its broad substrate spectrum and high tolerance to inhibitors in lignocellulosic hydrolysates [9,10]. However, a noteworthy phenomenon in oil-producing yeast during fermentation is its ability to switch between yeast and mycelial morphologies. This dimorphic transition directly impacts fermentation efficiency, as mycelial cells exhibit significantly lower lipid synthesis capacity than yeast cells. This is likely due to the fact that filamentous growth redirects cellular resources and energy towards morphological development rather than the accumulation of storage lipids [11]. Therefore, maintaining cellular stability in the high-yielding yeast-like morphology has become a critical scientific challenge for enhancing the oil production efficiency of this microorganism.

The morphology of microorganisms is regulated by multiple environmental factors, among which nutritional conditions represent one of the most critical influencing factors. For oil-producing yeasts, nitrogen source concentration has been extensively demonstrated as a core parameter regulating both cell growth and lipid accumulation [12,13]. When nitrogen sources in the medium are depleted, intracellular metabolic flux shifts, redirecting carbon sources toward lipid synthesis and storage—nitrogen source limitation-induced oleogenesis [14]. However, lower nitrogen sources are not always better: excessively low nitrogen source severely restricts cell growth, leading to insufficient biomass, while excessively high nitrogen levels inhibit the activity of key enzymes in lipid synthesis, redirecting carbon sources toward cell proliferation rather than lipid storage [15]. Therefore, determining an optimal nitrogen source range that balances cell growth and lipid accumulation is a primary prerequisite for achieving efficient fermentation. Therefore, determining the optimal nitrogen source concentration is the primary prerequisite for efficient fermentation. However, optimizing the nitrogen source alone may not suffice to achieve the theoretical maximum fermentation efficiency. Increasing evidence indicates that beyond nutritional conditions, the physicochemical environment—particularly dissolved oxygen (DO)—also plays a crucial role and may even directly influence cellular morphogenesis decisions [16,17].

Oxygen not only participates in energy metabolism but also serves as a direct substrate in fatty acid desaturation and triglyceride synthesis [18]. More importantly, recent studies indicate that dissolved oxygen supply may directly regulate the cellular morphology of *T. cutaneum*: under oxygen-limited conditions, this yeast readily undergoes a transformation from a yeast-like to a filamentous morphology, potentially representing an adaptive response to hypoxia stress [19]. The above studies indicate that, in addition to nitrogen source signaling, dissolved oxygen levels can serve as a relatively independent regulatory pathway that modulates lipid accumulation by controlling cellular morphological changes. Therefore, building upon the determination of optimal nitrogen source, further investigation into how enhanced dissolved oxygen supply can maintain the yeast-like morphology holds significant research value. However, these conventional strategies often involve a trade-off: they enhance oxygen transfer at the cost of increased energy consumption, detrimental shear forces, or oxidative stress on the cells [20,21]. Supplying enriched air may elevate partial pressure of oxygen but risks inducing oxidative stress, while pure oxygen use increases process costs and safety hazards. These drawbacks have prompted researchers to explore more economical and efficient oxygenation strategies.

As a core component of fermentation tanks, the structure of gas distributors directly influences the initial size and distribution of bubbles, thereby determining the volumetric mass transfer coefficient (*k*_L_a) and overall mixing performance of the tank [22]. Theoretically, a microporous ceramic membrane gas distributor (MCMGD) can generate smaller bubbles, significantly increasing the gas–liquid contact area. This enables efficient oxygen transfer without relying on high-intensity agitation. However, current research on the application of MCMGD in oil-producing yeast fermentation remains limited. In particular, systematic studies are lacking on whether they can regulate cell morphology through enhanced oxygen transfer, thereby increasing oil yield.

Based on our previous research on *T. cutaneum* B3, nitrogen source concentration appears to play a crucial role in inducing its biphasic transition [23]. Building upon this foundation, this study further investigates the effects of dissolved oxygen supply on the morphological transformation and lipid production of this strain. By introducing a MCMGD to enhance oxygen transfer and combining computational fluid dynamics (CFD) simulations to analyze its hydrodynamic mechanisms, we aim to establish the intrinsic relationship between reactor engineering and cellular morphology regulation.

## 2. Materials and Methods

### 2.1. Strain

Strain *T. cutaneum* B3 was obtained through combined mutagenesis of the parental strain *T. cutaneum* ACCC 20119 (originally from the former Soviet Union) using ethyl methanesulfonate and ultraviolet light, and has been deposited at the China Center for Type Culture Collection (CCTCC) under the accession number M2010076. For routine maintenance, the strain is preserved on yeast extract peptone dextrose agar slants (composition: 20 g/L glucose, 10 g/L yeast extract, 10 g/L peptone, 20 g/L agar; pH adjusted to 5.8–6.0), while long-term cryopreservation employs 25% (*v*/*v*) sterilized glycerol suspensions stored at −80 °C. The strain undergoes monthly activation through a standardized protocol: primary incubation at 28–30 °C for 48 h in liquid culture, followed by secondary preservation at 4 °C on solid medium.

### 2.2. Extended Fermentation Culture

The strain amplification protocol comprises four standardized stages:

1. Stage a: Inoculate strain B3 on yeast extract peptone dextrose agar slants (composition as above) and incubate at 28 °C for 3–5 days.
2. Stage b: Transfer isolated colonies to 250 mL flasks containing 60 mL liquid medium. Culture at 28 °C with 200 rpm agitation for 36–48 h.
3. Stage c: Inoculate 10 mL starter culture into 500 mL flasks with 120 mL fermentation medium (composition: 60 g/L glucose, 0.75 g/L KH_2_PO_4_, 0.4 g/L CaCl_2_∙2H_2_O, 0.4 g/L MgSO_4_∙7H_2_O; pH adjusted to 5.8–6.0). Maintain identical temperature and agitation conditions. Monitor biomass, nitrogen source consumption, glucose utilization, lipid production, and cellular morphology through periodic sampling.
4. Stage d: Scale-up fermentation was conducted in a 5.0 L bioreactor (Biotech-5BG-4, Shanghai Baoxing Bio-Engineering Equipment Co., Ltd. (BXBIO), Shanghai, China) with the following system configurations:

Reactor design: Jacket-cooled vessel with dual baffle plates; magnetically coupled drive system featuring a single-layer six-blade Rushton turbine. Operational parameters: Working volume: 2.0 L; inoculum: 6% (*v*/*v*) cell suspension (5 × 10^6^ cells/mL); initial conditions: 200 rpm agitation, 3.0 L/min aeration (1.5 vvm), pH 5.8–6.0; pH control: automated titration with 8 M NaOH (setpoint > 4.0); monitoring regime: hourly quantification of DO, pH, and metabolite profiles.

### 2.3. Enhanced DO Fermentation Condition

We systematically investigated strain growth characteristics, glucose–nitrogen substrate consumption patterns, lipid biosynthesis efficiency, and DO profiles under controlled aeration rates (0.5–2.5 vvm), agitation speeds (200–320 rpm) and oxygen-enriched air supply. Three nitrogen gradient media were established, Nitrogen-Limited Medium (NLM), Nitrogen-Moderate Medium (NMM), and Nitrogen-Rich Medium (NRM), containing 1.0 g/L, 3.0 g/L, and 5.0 g/L yeast extract, respectively. During oxygen-enrichment phases (1.0–5.0 g/L O_2_), baseline operational parameters (200 rpm agitation, 1.0–2.0 vvm aeration) were maintained to prevent DO depletion below detectable levels.

Comparative evaluation of aeration systems was conducted using two configurations: conventional air distributor (CK) and optimized MCMGD (40 mm × 1.6 mm). Parallel fermentation trials were initiated with 150 rpm magnetic stirring under variable aeration rates (0.5, 0.8, 1.0, and 1.2 vvm), while CK controls maintained a fixed 1.0 vvm aeration. All batches contained identical initial nutrient loads: 3.0 g/L yeast extract (nitrogen source) and 60 g/L glucose. Fermentation termination criteria included near-complete sugar exhaustion (<1.0 g/L detectable reducing sugars), followed by bioreactor harvest.

### 2.4. Strain Growth Morphology

Cellular morphology analysis was performed using digital optical microscopy (Ted Pella, Inc., Redding, CA, USA; 3-megapixel integrated camera) interfaced with Motic Images Plus 2.0 software. Yeast samples from shake-flask and bioreactor cultures were stained with Methylene Blue trihydrate (Aladdin™ M102717, 90% purity, Shanghai Aladdin Biochemical Technology Co., Ltd., Shanghai, China) and imaged under ×1000 magnification with oil immersion optics.

Remove the MCMGD from the fermentation tank, rinse it with ultrapure water, and dry it overnight in a 60 °C oven. Transfer it to a vacuum drying oven for further drying. Using tweezers, remove the sample and affix it to the back of a cylindrical sample holder with conductive tape. After gold coating, examine it using a scanning electron microscope (S-3400N, Hitachi, Ltd., Tokyo, Japan).

### 2.5. Determination of Biomass, Oil, Total Nitrogen, and Glucose

The fermentation broth underwent primary separation through centrifugation (10,000 rpm, 10 min) to partition cellular biomass from supernatant. Harvested cells were washed three times with sterile deionized water and quantified via dry cell weight (DCW) determination.

The cells were then mechanically disrupted using a sterile mortar under liquid nitrogen cooling. The lysate was subjected to acid hydrolysis using 4 M HCl (6 mL/g DCW). After vortex mixing, the mixture was left to stand at 25 °C for 30 min. Thermal lysis was enhanced through boiling water bath incubation (10 min) followed by immediate quenching in an ice bath. Lipid extraction employed a chloroform:methanol (2:1 *v*/*v*) solution (20 mL) with intermittent agitation over 30 min. Finally, centrifuge the sample (10,000 rpm, 5 min), use a pipette to transfer the chloroform phase into a small round-bottom flask, and concentrate it under reduced pressure using a rotary evaporator until the solvent is completely removed. Weigh the oily residue collected at the bottom of the flask [24].

The supernatant obtained by centrifugation was utilized for glucose and total nitrogen quantification. In single carbon source glucose fermentation systems, glucose concentration was determined using the 3,5-dinitrosalicylic acid (DNS) method [25]. Total nitrogen content was determined according to the Kjeldahl method specified in the Chinese National Standard GB 5009.5-2010 [26].

### 2.6. CFD Simulation Analysis

Table 1 summarizes the bioreactor operating parameters. Using the literature as a reference, computational fluid dynamics simulations were performed to analyze the flow patterns within the original reactor and the bioreactor equipped with a microporous membrane air distributor [27,28]. SolidWorks 3D models (v.2013) were constructed for two configurations: the standard bioreactor and its membrane-aerated variant. Numerical analysis combined the multi-reference frame (MRF) approach with Eulerian multiphase modeling and population balance methods to characterize gas–liquid hydrodynamics.

To optimize computational efficiency, the porous membrane structure was simplified as a planar surface with 300 evenly spaced micropores. ICEMCFD v5.0 generated hybrid meshes containing over 15 million elements: hexahedral for the fluid domain and tetrahedral for the impeller region. Governing equations were solved using ANSYS CFX 14.0’s coupled multi-grid algorithm to determine mass transfer coefficients [27].

### 2.7. Determination of k_L_a

*k*_L_a was determined using the dynamic gassing-in method. Prior to experimentation, the DO electrode underwent 6–12 h electrical polarization followed by zero-point calibration with saturated Na_2_SO_3_ solution. A 2.0 L sterilized fermentation medium (NMM containing 3.0 g/L yeast extract) was prepared under atmospheric pressure conditions, maintaining identical operational parameters (agitation speed, aeration rate, temperature, and initial pH) to batch fermentation processes, but without yeast inoculation, to specifically measure *k*_L_a in microorganism-free culture conditions. Following 12 h of system stabilization, full-scale DO calibration was performed (slope value set to 100%). Subsequent experimental procedures included (1) closing the air inlet while introducing nitrogen to purge residual oxygen until stable DO baseline (0% to negative values) was achieved; (2) initiating aeration at a fixed rate (1.0 vvm, 2.0 L/min) with simultaneous DO monitoring; and (3) commencing time recording when DO reached 0%, with data acquisition every 10 s until DO stabilized at 100% or reached equilibrium. The *k*_L_a value was calculated using the following first-order kinetic model:

$$\ln\frac{\left(100-DO\right)}{100}=k_{L}a*t$$

where DO represents dissolved oxygen percentage (%) and t denotes aeration time.

## 3. Results and Discussion

### 3.1. Oil Production Capacity at Different Rotational Speeds and Aeration Rates

**Figure 1:**
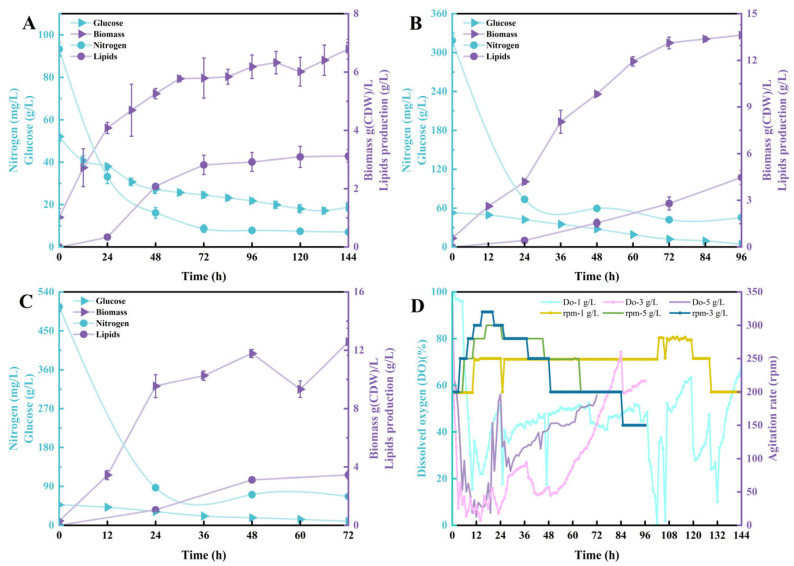
Consumption of glucose and total nitrogen and accumulation of biomass and lipids during the batch culture of *T. cutaneum* B3 within the fermenter using media supplemented with 60 g/L glucose and yeast extract at 1.0 g/L (**A**), 3.0 g/L (**B**), or 5.0 g/L (**C**), respectively, and the dissolved oxygen (DO) profiles of the culture and agitation rate applied to the process (**D**). Data are presented as mean ± SD from three independent biological replicates (n = 3).

During microbial fermentation for oil production, DO levels are a key factor influencing cell growth and lipid accumulation [29]. Therefore, batch fermentation was conducted by maintaining DO levels above 5% in the fermenter through the addition of yeast extract at varying initial concentrations and continuous adjustment of stirring speed. Experimental results indicate that when fermenting with 1 g/L nitrogen source for 144 h, the final yeast dry weight reached only 6.78 ± 0.34 g/L, with oil content at 3.12 ± 0.22 g/L and oil concentration approximately 46.02% (Figure 1A). Notably, glucose utilization began to decline after 48 h. At 144 h, 18.92 g of glucose remained from the initial feed, accounting for approximately 26.31% of the initial amount. The excessively high residual sugar concentration in the fermentation broth correlated with the substrate’s total nitrogen source content. At 48 h of fermentation, the total nitrogen level dropped to 16 mg/L, resulting in insufficient nitrogen supply for yeast growth. This impaired biomass accumulation and glucose consumption [30]. Lipid accumulation also gradually stabilized after 48 h. Throughout this fermentation, the low cell growth rate resulted in minimal DO demand, with DO levels maintained above zero at a maximum agitation speed not exceeding 320 rpm (Figure 1D). Concurrently, we observed cell morphology at 12-h intervals. Cells within the fermenter exhibited uniformly short, single-cell morphology consistent with that observed in conventional fixed-speed fermentation (Figure S1).

When the nitrogen source concentration was increased to 3 g/L, it was rapidly depleted within 24 h, dropping to 70 mg/L. Correspondingly, the cells’ demand for DO increased. During this phase, the rotation speed was increased to 320 rpm, and the aeration rate was also increased to approximately 4.5 min/L (2.25 vvm) (Figure 1B). Over the subsequent 72 h, the nitrogen source decreased to 40 mg/L, while the strain depleted the supplied glucose. Under these conditions, the rate at which this strain utilizes glucose remains relatively low. The final dry cell weight achieved through enhanced DO and nitrogen source supply was only 13.62 ± 0.66 g/L, with an oil content of 4.47 ± 0.43 g/L, corresponding to a concentration of approximately 32.82%. This outcome is likely attributed to excessive agitation caused by the increased stirring speed, coupled with the adverse effects of elevated aeration on cell growth within the fermenter. Microscopic observations also indicated that during phases with moderate agitation and aeration rates, cell morphology showed no significant difference compared to the 1 g/L concentration. Notably, during the early fermentation stage (12–24 h) with higher DO demand, slight variations in individual cell length were observed, with slightly elongated cells, though this phenomenon rarely occurred in the later fermentation phase (Figure S1).

When nitrogen source supply was increased to 5 g/L, the agitation speed was raised to 200–320 rpm and the aeration rate increased to 5.0 min/L (2.5 vvm) during the early fermentation stage to maintain DO supply. Cells depleted glucose within 72 h, significantly improving the utilization efficiency of both total nitrogen and glucose (Figure 1C). However, the final cell dry weight was only 12.58 ± 0.93 g/L, with lipid content at 3.44 ± 0.25 g/L, corresponding to a concentration of approximately 27.34%. After further increasing the nitrogen source concentration, neither the yeast biomass nor the lipid content showed any further increase. This may be because the nitrogen source concentration had exceeded the lipid production threshold at this point, no longer promoting the accumulation of biomass or lipids. Regarding cell morphology, dynamic changes during fermentation resembled those observed at 3 g/L nitrogen source supply. However, due to excessive agitation speed, although microscopic analysis showed no signs of hyphal breakage, it remains difficult to rule out damage to hyphal cells from intense disturbances such as mechanical shear forces (Figure S1). In summary, under varying nitrogen source concentrations, adjusting the agitation speed and enhancing the aeration rate effectively maintained DO supply while preserving the uniform single-cell morphology of the test strain *T. cutaneum* B3 throughout fermentation stages. The highest lipid yield was achieved when the initial nitrogen source concentration was set at 3 g/L.

### 3.2. Lipid Production Capacity of Oxygen-Enriched Air Fermentation Strains

**Figure 2:**
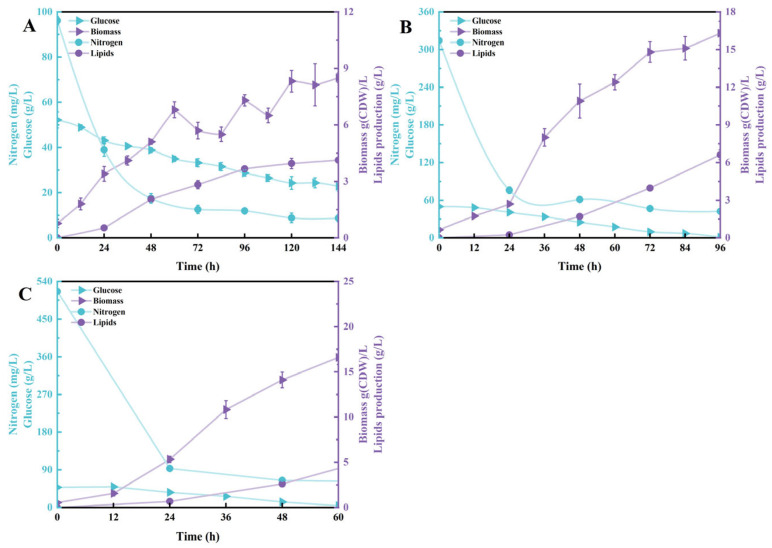
Effect of supplementation with oxygen-enriched air on the consumption of glucose and total nitrogen and accumulation of biomass and lipids during the batch culture of *T. cutaneum* B3 with media composed of 60 g/L glucose and yeast extract supplemented at 1.0 g/L (**A**), 3.0 g/L (**B**), or 5.0 g/L (**C**). Data are presented as mean ± SD from three independent biological replicates (n = 3).

In addition to enhancing oxygen mass transfer by increasing the stirring speed and aeration rate, directly raising the partial pressure of oxygen in the feed gas is also an effective method to improve oxygen supply during fermentation [31]. Using a three-way valve, we introduced a mixture of air and pure oxygen into the fermenter. With an initial nitrogen source loading of 1 g/L, the fermentation process lasted 144 h, yielding residual sugar levels as high as 22.8 ± 2.12 g/L, with total nitrogen source consumption reaching 99.1%. The final fermentation produced a dry cell biomass of 8.5 ± 0.25 g/L and lipid content of 4.13 ± 0.12 g/L (Figure 2A). Compared to conditions with increased rotation speed and aeration rate, this strategy yielded slightly higher dry cell weight and lipid production, though the differences were not significant. This result further confirms that under low-nitrogen source conditions, nitrogen limitation is the primary bottleneck factor constraining lipid synthesis, with its impact on metabolic flux far exceeding that of dissolved oxygen levels [18]. Therefore, under low nitrogen source conditions—regardless of enhanced DO supply—both cell biomass and lipid accumulation remained low due to insufficient nitrogen sources for synthesizing proteins and nucleic acids required for cell proliferation [30]. Cell morphology consistently exhibited high-yield yeast-like cells (Figure S2). Although cell morphology remains stable under this strategy, the low biomass concentration renders it unsuitable as the initial inoculum for batch fermentation.

After introducing oxygen-enriched air, increasing the initial nitrogen source concentration to 3 g/L resulted in glucose utilization efficiency consistent with the increased rotation speed strategy, with glucose depletion occurring at 96 h. At 96 h, residual total nitrogen source content was 42.23 ± 1.76 mg/L, cell dry weight increased to 16.3 ± 0.95 g/L, and oil yield rose to 6.61 ± 0.38 g/L (Figure 2B). Compared to our previous studies using only fixed agitation speeds and aeration rates, the addition of oxygen-enriched air improved total nitrogen utilization efficiency in the early fermentation stage but prolonged fermentation time and reduced biomass dry weight, lipid yield, and concentration [23]. When nitrogen source concentration was further increased to 5 g/L, compared to increasing rotation speed, the introduction of oxygen-enriched air shortened fermentation time, allowing glucose to be largely depleted within approximately 60 h. Post-fermentation, total nitrogen source concentration decreased to 61.13 ± 1.76 mg/L, with cell dry weight reaching 16.6 ± 0.79 g/L, significantly enhancing both the cell growth rate and nitrogen source utilization efficiency. However, lipid content and concentration were slightly lower than the 3 g/L treatment under the same strategy, at 6.04 ± 0.28 g/L and 36.39% (Figure 2C). It is speculated that this may be related to shifts in metabolic regulation under high-nitrogen conditions. Previous studies have reported that an excess supply of nitrogen sources can induce fluctuations in intracellular energy status, maintaining AMP concentrations at relatively high levels, which in turn sustains or enhances the allosteric activity of mitochondrial NAD^+^-dependent isocitrate dehydrogenase (NAD^+^-IDH). The sustained activation of this enzyme accelerates the oxidative degradation of isocitrate, causing the dynamic equilibrium between citrate and isocitrate in the mitochondrial matrix to shift toward degradation. This inhibits the accumulation of citrate and its transport and export to the cytoplasm, ultimately weakening the supply of precursors for fatty acid synthesis [14].

Notably, during the early fermentation stage under the oxygen-enriched air strategy, microscopic morphology analysis revealed the emergence of distinct mycelial-type cells, with a higher proportion observed compared to the increased agitation speed strategy (Figure S2). This phenomenon may be related to the local microenvironment during the initial fermentation stage: in the early phase of oxygen-enriched air introduction, mixed mass transfer efficiency may be less thorough than under high-speed agitation, leading to local oxygen concentration gradients or nutrient imbalances that induce cellular morphological differentiation [19]. Research indicates that oleaginous yeasts undergo morphological transitions from yeast to hyphal forms under certain environmental stresses, typically associated with regulatory changes in cell wall synthesis genes [32,33]. Overall, while the oxygen-enriched air strategy demonstrated superior lipid content compared to increased agitation and aeration, the method and timing of enhanced DO supply are critical for maintaining a single yeast morphology. Both strategies confirm that adequate dissolved oxygen is a key prerequisite for stable cell morphology and efficient oil production.

### 3.3. Applications of MCMGD

In our previous study [23], the optimal initial nitrogen source concentration for *T. cutaneum* B3 fermentation was determined to be 3 g/L. Under these conditions, the dry biomass of the fungal cells reached 27.1 g/L, with lipid content attaining 12.5 g/L. This study demonstrated that maintaining DO supply plays a crucial role in regulating cell morphology through strategies such as adjusting rotation speed, aeration rate, and introducing oxygen-enriched air. However, compared to the previous fermentation strategy, both cell dry weight and oil yield were significantly lower under the aforementioned operating conditions. Although these discrepancies stem from variations in agitation speed, DO supply rate, and aeration volume, the increased mechanical shear stress and heat accumulation induced by high agitation speeds also adversely affect biomass and oil accumulation while elevating energy consumption and costs. Consequently, this study explored replacing the fermenter’s air distributor to enhance oil yield while intensifying DO supply and preserving cell morphology, thereby investigating more economically viable fermentation strategies.

**Figure 3:**
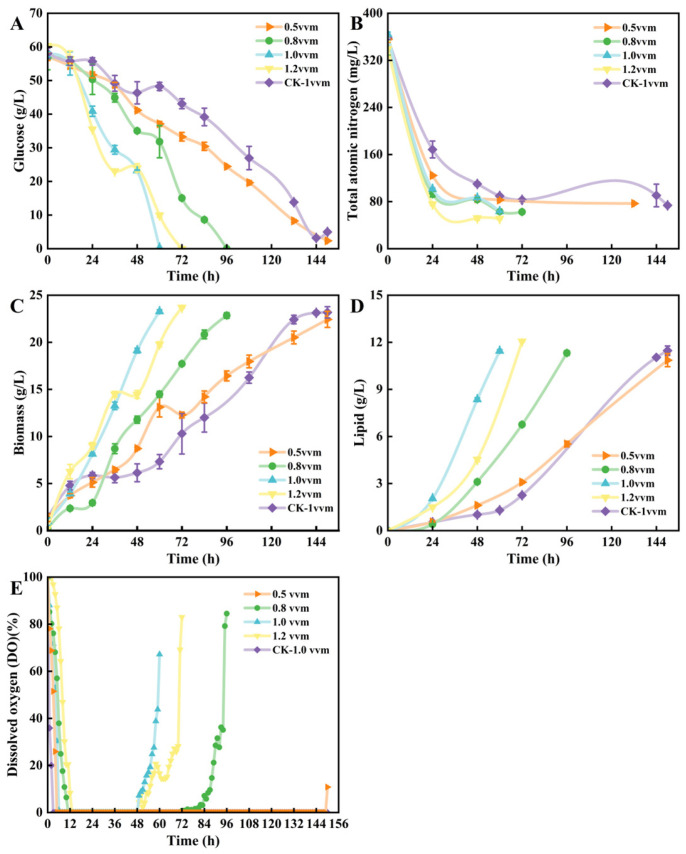
Time courses for the consumption of glucose (**A**) and total nitrogen (**B**) and the accumulation of biomass (**C**) and lipids (**D**) as well as the DO profile (**E**) during the batch culture of *T. cutaneum* B3 in the bioreactor equipped with microporous ceramic membrane gas distributor (MCMGD) under different aeration rates. The fermenter was operated at 150 rpm using media supplemented with 60 g/L glucose and 3.0 g/L yeast extract. The CK bioreactor was operated at 150 rpm, 1.0 vvm equipped with conventional perforated ring gas distributors. Data are presented as mean ± SD from three independent biological replicates (n = 3).

MCMGD generate finer, more uniform bubbles, significantly increasing the gas–liquid contact area and enhancing the *k*_L_a. This achieves higher dissolved oxygen transfer efficiency at lower aeration rates [34]. Figure 3 demonstrates the fermentation performance of the MCMGD. At half the aeration rate, the new strategy exhibited fermentation progress comparable to the original distributor, both completing fermentation with glucose depletion at 150 h. However, at aeration rates of 1.0 vvm and above, the new strategy significantly shortened the fermentation cycle to 60–96 h (Figure 3C). Specifically, the new strategy yielded 11.44 ± 0.09 g/L oil at 60 h under 1.0 vvm, 12.06 ± 0.04 g/L at 72 h under 1.2 vvm, and 11.31 ± 0.15 g/L at 96 h under 0.8 vvm; the original distributor and 0.5 vvm conditions yielded 11.47 ± 0.29 g/L and 10.86 ± 0.41 g/L at 150 h, respectively (Figure 3B). At different aeration rates, the final dry biomass yield obtained with the new strategy was comparable to that of the original distributor (Figure 3A), indicating that the MCMGD significantly shortened the fermentation cycle without sacrificing biomass accumulation, thereby substantially improving production efficiency.

DO supply is closely correlated with total nitrogen utilization efficiency. In this study, the new strategy yielded higher total nitrogen utilization efficiency and stronger DO supply. With the original distributor, DO dropped to zero within 3 h after fermentation initiation; under the new strategy at 0.5 vvm, DO subsequently fell to zero and remained there. At 6 h into fermentation, DO began dropping to zero under the 1.0 vvm condition, followed sequentially by the 0.8 vvm and 1.2 vvm conditions. However, all three conditions showed recovery between 48 and 72 h (Figure 3E). Although both the new and old strategies experienced DO drops to zero during fermentation, the duration of the drop was significantly shorter under the new strategy. Under the 1.0 vvm aeration rate condition, both cell biomass and oil production intensity reached their highest levels, achieving a fermentation productivity of 0.191 g/(L·h) and a yield of 0.199 g/g. Although slightly lower than the yield under 200 rpm and 1.5 vvm conditions in previous studies, this condition exhibited the highest production rate among the different DO enhancement strategies, increasing by 1.47-fold and 2.16-fold, respectively, and oil content increased to 49.16% (Table 2).

Cell morphology is a key factor influencing high oil yield in *T. cutaneum* B3. Compared to the original air distributor, the MCMGD demonstrated superior performance in maintaining yeast-like cell morphology. Significant biphasic transformation occurred in the fermentation tank using the original distributor, with hyphal-type cells predominating throughout the fermentation process (Figure S3). This may be related to oxygen deficiency caused by premature oxygen depletion. Under the new strategy, except at 0.5 vvm, *T. cutaneum* B3 cells maintained a single yeast-type morphology throughout the fermentation period at all other aeration rates.

In summary, the MCMGD effectively enhances DO supply during fermentation while reducing both rotation speed and aeration rate, maintaining *T. cutaneum* B3 cells in the ideal high-yield yeast morphology. The optimized fermentation parameters are: aeration rate 1.0 vvm, rotation speed 150 rpm, pH 5.0–6.0, temperature 28 °C, and initial inoculum 8–10%.

### 3.4. Mechanism by Which Membrane Air Distributors Enhance Oil Fermentation Yield

To investigate the performance advantages of MCMGD, further studies were conducted on mass transfer and mixing processes in fermenters using both types of diffusers. The original ring-shaped perforated gas distributor features 26 large-diameter vent holes measuring 1.0 mm (Figure S4A). After aeration, bubbles tend to aggregate and adhere at the outlet, leading to further enlargement of bubble size and a shortened residence time in the liquid phase, which is detrimental to microbial oxygen utilization. SEM observations revealed that the MCMGD surface is densely covered with cavities approximately 500 nm in diameter (Figure S4B–D), which is expected to generate smaller bubbles. Even if bubbles aggregate, large bubbles are less likely to form, thereby prolonging the residence time of oxygen-containing microbubbles in the liquid phase. Research indicates that bubble diameter is a key parameter affecting gas–liquid mass transfer efficiency. Smaller bubbles possess a larger specific surface area, significantly enhancing the *k*_L_a [35,36]. During aerobic microbial fermentation, oxygen transfer from the gas phase to the liquid phase is equally critical alongside nutrient mass transfer, directly impacting dissolved oxygen supply and gas–liquid mixing efficiency [37]. Under a 1.0 vvm aeration rate, the *k*_L_a was measured for fermenters equipped with either a MCMGD or the original distributor. The results showed that the microporous membrane group had a *k*_L_a of 0.012 s^−1^, approximately 2.2 times that of the original group (0.0055 s^−1^), indicating significantly enhanced DO supply and transfer performance after modification.

**Figure 4:**
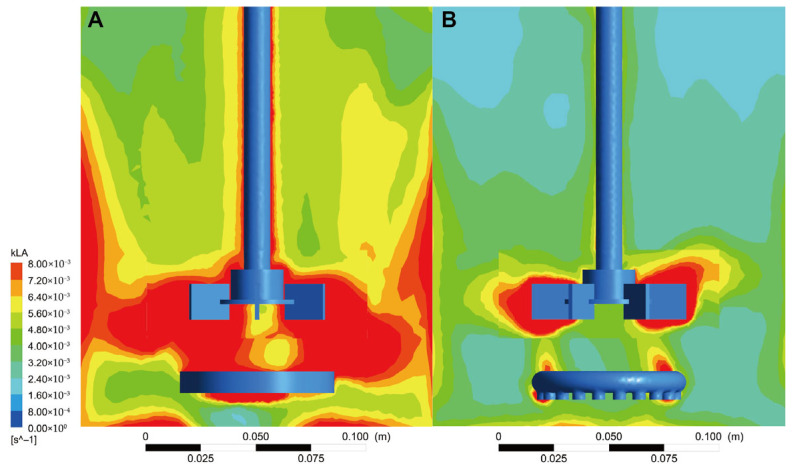
The volumetric oxygen transfer coefficient (*k*_L_a) profile within the bioreactor equipped with the MCMGD (**A**) and perforated ring gas distributors (**B**). Color gradient represents the *k*_L_amagnitude, while red denotes the highest and dark blue denotes the lowest.

CFD simulations were conducted to analyze *k*_L_a, tank mixing and flow field distribution, DO content, and bubble size at the gas outlet under two different distributor configurations. This study elucidates the mechanism by which MCMGDs enhance fermentation performance from a fluid dynamics perspective. Simulation results are represented by color gradients: deeper red areas indicate higher *k*_L_aand more efficient DO transfer, while larger blue areas denote lower *k*_L_a and restricted DO transfer. In the fermenter equipped with the MCMGD, the area of deep red zones was significantly larger than in the original tank. These zones extended not only near the agitator but also to both sides of the tank, while the area of light blue dead zones was markedly reduced (Figure 4). The results indicate that the membrane air distributor enhances DO supply within the tank, enabling rapid and efficient oxygen transfer during aerobic fermentation. This supports the rapid growth and lipid accumulation of *T. cutaneum* B3 cells.

**Figure 5:**
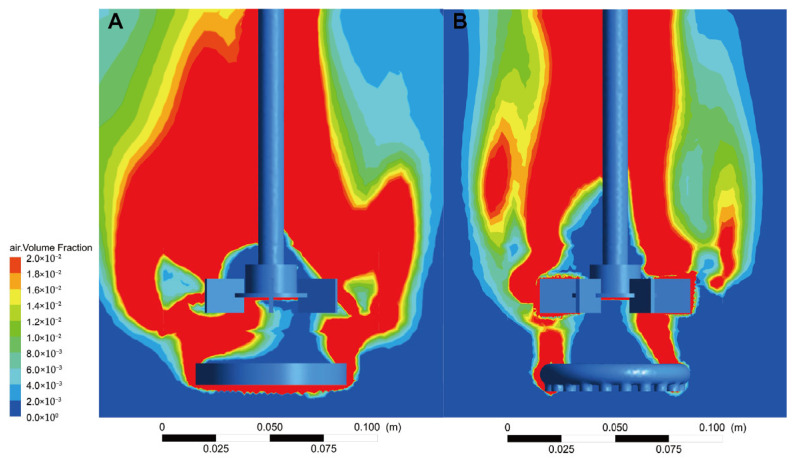
The gas holdup distribution within the bioreactor equipped with the MCMGD (**A**) and perforated ring gas distributor (**B**). Red denotes the highest gas holdup and dark blue denotes the lowest.

The CFD simulation with the membrane air distributor yielded an average *k*_L_a value of 0.0097 s^−1^, approximately three times that of the original tank (0.0035 s^−1^). This result closely matches the measured value, validating the reliability of the CFD model. Gas content simulation results indicate more uniform gas distribution within the membrane diffuser tank. The red high-gas-content zone is broader, extending not only axially along the agitator shaft but also radially toward the edge regions, with fewer light blue dead zones. In contrast, the original tank exhibits gas concentration primarily near the agitator shaft (Figure 5B). Simulation calculations indicate that the average bubble diameter in the original tank is 2.54 mm, significantly larger than the 1.47 mm under membrane distributor conditions. This demonstrates that the MCMGD generates smaller bubbles and achieves broader axial and radial distribution, thereby enhancing DO supply efficiency (Figure 5A). Liu et al. noted that uneven gas distribution frequently occurs in small-to-medium-sized fermenters. Increasing turbulence intensity and rotation speed provides enhanced shear forces for bubble breakup, which improves gas–liquid mass transfer and promotes more uniform bubble distribution within the vessel [38].

**Figure 6:**
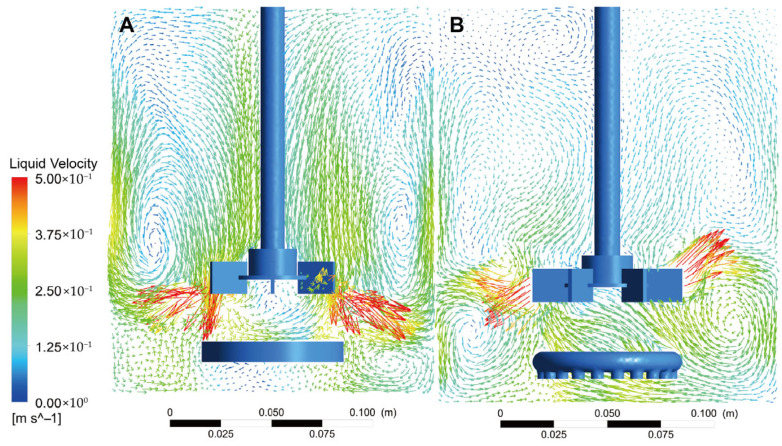
Liquid velocity field within the bioreactor equipped with the MCMGD (**A**) and perforated ring gas distributor (**B**).

Although the average liquid velocity obtained from CFD simulations under both distributor conditions was 0.16 m/s, their flow field distributions differed. Both exhibited the most thorough mixing and dense flow fields in the agitator blade regions. However, the MCMGD generated axial lift due to its densely distributed gas-emitting pores, which improved overall fluid mixing within the tank (Figure 6A). In contrast, the liquid flow field within the original annular distributor primarily concentrated in the radial region influenced by the agitator, with relatively limited axial flow distribution (Figure 6B). Effective axial mixing is crucial for ensuring uniform distribution of nutrients and dissolved oxygen within the reactor [39,40].

In summary, the MCMGD effectively enhances oxygen transfer rates within the reactor during *T. cutaneum* B3 oil fermentation while operating at lower agitation speeds and airflow volumes, thereby strengthening DO supply. Its densely packed microporous surface generates smaller and more numerous bubbles. As these bubble clusters ascend, they mix thoroughly with the transverse radial flow created by agitation, prolonging the residence time of oxygen-rich microbubbles within the liquid phase. This promotes mass transfer and mixing between the substrate and gas–liquid phases, supporting efficient cellular metabolism. Therefore, adequate DO supply and uniform gas–liquid mixing are critical prerequisites for achieving high-yield yeast-type fermentation growth with *T. cutaneum* B3.

## 4. Conclusions

This study investigated the effects of varying initial nitrogen source concentrations and dissolved oxygen enhancement strategies on the fermentation process of *T. cutaneum* B3. The results indicated that under conditions of 3 g/L yeast extract addition, 150 rpm agitation speed, and 1.0 vvm aeration rate, the MCMGD significantly improved oxygen transfer efficiency in the fermenter, effectively enhancing DO supply. This strategy successfully maintained *T. cutaneum* B3 in a high-yield yeast morphology, thereby increasing oil yield. It clarified the critical role of gas distributor optimization in linking cell morphology regulation to lipid synthesis. Although the current strategy demonstrates clear advantages in optimizing lipid fermentation in small-scale fermenters, further validation of the distributor’s feasibility and stability under actual industrial conditions at pilot or scale-up levels is required. Overall, this study reveals the pivotal role of gas distributor optimization in regulating filamentous yeast morphology and enhancing lipid accumulation, providing novel strategic insights for improving microbial lipid yields through fermentation engineering approaches.

## Figures and Tables

**Table 1:** Parameters of the bioreactor.

Parameters	Value
Total volume	5.0 L
Working volume	2.0 L
Liquid height	92 mm
Tank diameter	200 mm
Baffle length	160 mm
Baffle width	12 mm
Diameter of the 6-straight blade disk turbine impeller	80 mm
Blade length	20 mm
Blade width	15 mm
Diameter of the ring gas sparger	65–75 mm
Diameter of the ceramic membranous gas distributor	40 mm
Thickness of the ceramic membranous gas distributor	1.6 mm
Diameter of the inlet pipeline	5 mm
Distance of the impeller from the reactor bottom	35 mm

**Table 2:** Lipid production and cell growth of *T. cutaneum* B3 under different nutrition and culture modes.

Operation Mode	Gas Distributors	Agitation Rate (rpm)	Aeration Rate (vvm)	Lipid Content (*w*/*w*, %)	Biomass Productivity (g/L/h)	Lipid Productivity (g/L/h)	Lipid Yield (g/g)
1.0 g/L-rpm ^a^	R	200–280	1.0~1.5	46.02	0.047	0.022	0.094
3.0 g/L-rpm ^a^	R	200–320	1.0~2.25	32.82	0.142	0.047	0.092
5.0 g/L-rpm ^a^	R	200–300	1.0~2.5	27.42	0.175	0.048	0.089
1.0 g/L-O_2_^b^	R	200	1.0~1.5	48.59	0.059	0.029	0.141
3.0 g/L-O_2_^b^	R	200	1.0~2.0	40.61	0.170	0.069	0.135
5.0 g/L-O_2_^b^	R	200	1.0~2.0	36.39	0.277	0.101	0.138
CK ^c^	R	150	1.0	47.73	0.161	0.077	0.201
0.5 vvm ^d^	M	150	0.5	48.42	0.150	0.072	0.198
0.8 vvm ^d^	M	150	0.8	49.54	0.238	0.118	0.199
1.0 vvm ^d^	M	150	1.0	49.16	0.388	0.191	0.199
1.2 vvm ^d^	M	150	1.2	50.89	0.329	0.168	0.200

^a,b^ Yeast extract was supplemented at 1.0 g/L, 3.0 g/L, or 5.0 g/L, respectively; ^c,d^ yeast extract was supplemented at 3.0 g/L; ^a^ agitation rate and aeration rate were adjusted accordingly; ^b^ oxygen-enriched air supplementation mode was applied; R—conventional perforated ring gas distributors; M–microporous ceramic membrane gas distributor.

## Data Availability

The original contributions presented in this study are included in the article/Supplementary Material. Further inquiries can be directed to the corresponding author.

## Supplementary Materials

The following supporting information can be downloaded at: https://www.mdpi.com/article/10.3390/jof12050312/s1. Figure S1: Effect of agitation rate and aeration on the morphology of *T. cutaneum* B3, which was observed under magnification of 1000 for culture with media composed of 60 g/L glucose and yeast extract supplemented at 1.0 g/L (1 g/L-rpm), 3.0 g/L (3.0 g/L-rpm) or 5.0 g/L (5.0 g/L-rpm). The bars represent 30 μm; Figure S2: Effect of supplement with oxygen-enriched air on the morphology of *T. cutaneum* B3, which was observed under magnification of 1000 for culture with media composed of 60 g/L glucose and yeast extract supplemented at 1.0 g/L (1.0 g/L-O_2_), 3.0 g/L (3.0 g/L-O_2_) or 5.0 g/L (5.0 g/L-O_2_). The bars represent 30 μm; Figure S3: Effect of aeration rate on the morphology of *T. cutaneum* B3 during the batch culture in the bioreator equipped with microporous ceramic membrane gas distributor (MCMGD). The morphology was observed under magnification of 1000 for culture with media composed of 60 g/L glucose and 3.0 g/L yeast extract. The bars represent 30 μm. CK bioreactor was operated at 150 rpm, 1.0 vvm equipped with conventional perforated ring gas distributors; Figure S4: Perforated ring gas distributor (A) and MCMGD (B). C and D represents the scanning electron microscopy (SEM) image of MCMGD.

## Abbreviations

The following abbreviations are used in this manuscript:

DO	Dissolved oxygen
CFD	Computational fluid dynamics
CCTCC	China Center for Type Culture Collection
NLM	Nitrogen-limited medium
NMM	Nitrogen-moderate medium
NRM	Nitrogen-rich medium
DCW	Dry cell weight
MRF	Multi-reference frame
DNS	3,5-dinitrosalicylic acid
MCMGD	Microporous ceramic membrane gas distributor
*k*_L_a	Volumetric mass transfer coefficient
