# Supplementary material for: Morphological Shift and Lipid Accumulation in Trichosporon cutaneum B3 Induced by Enhanced Dissolved Oxygen
Source: J Fungi (Basel). 2026 Apr 24;12(5):312. doi: 10.3390/jof12050312 (PMC13207652; doi:10.3390/jof12050312)
Supplement: Supplementary file 1 [file jof-12-00312-s001.zip › jof-4216352-supplementary.pdf]

Supplemental Figure legends

Figure S1

Effect of agitation rate and aeration on the morphology of *T. cutaneum* B3, which was observed under magnification of 1000 for culture with media composed of 60 g/L glucose and yeast extract supplemented at 1.0 g/L (1 g/L-rpm), 3.0 g/L (3.0 g/L-rpm) or 5.0 g/L (5.0 g/L-rpm). The bars represent 30 μm.

Figure S2

Effect of supplement with oxygen-enriched air on the morphology of *T. cutaneum* B3, which was observed under magnification of 1000 for culture with media composed of 60 g/L glucose and yeast extract supplemented at 1.0 g/L (1.0 g/L-O_2), 3.0 g/L (3.0 g/L-O_2) or 5.0 g/L (5.0 g/L-O_2). The bars represent 30 μm.

Figure S3

Effect of aeration rate on the morphology of *T. cutaneum* B3 during the batch culture in the bioreator equipped with microporous ceramic membrane gas distributor (MCMGD). The morphology was observed under magnification of 1000 for culture with media composed of 60 g/L glucose and 3.0 g/L yeast extract. The bars represent 30 μm. CK bioreactor was operated at 150 rpm, 1.0 vvm equipped with conventional perforated ring gas distributors.

Figure S4

Perforated ring gas distributor (A) and MCMGD (B). C and D represents the scanning electron microscopy (SEM) image of MCMGD.

Figure S1

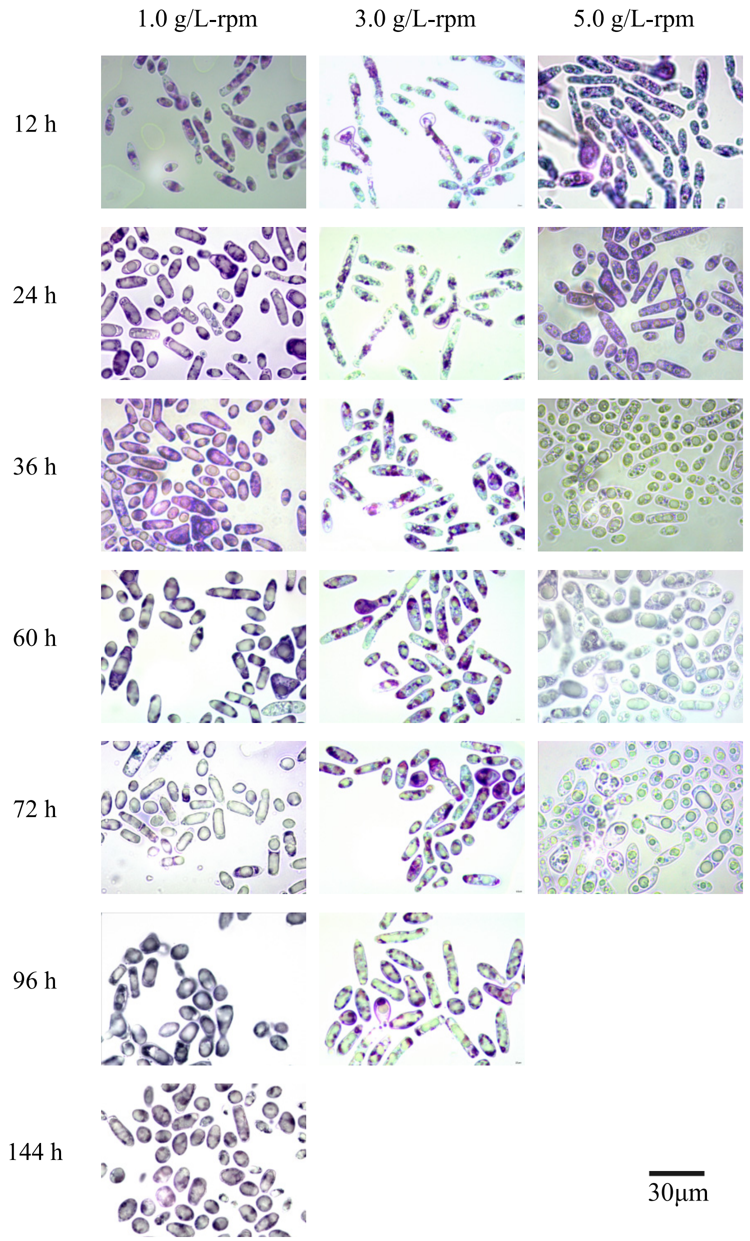

Figure S2

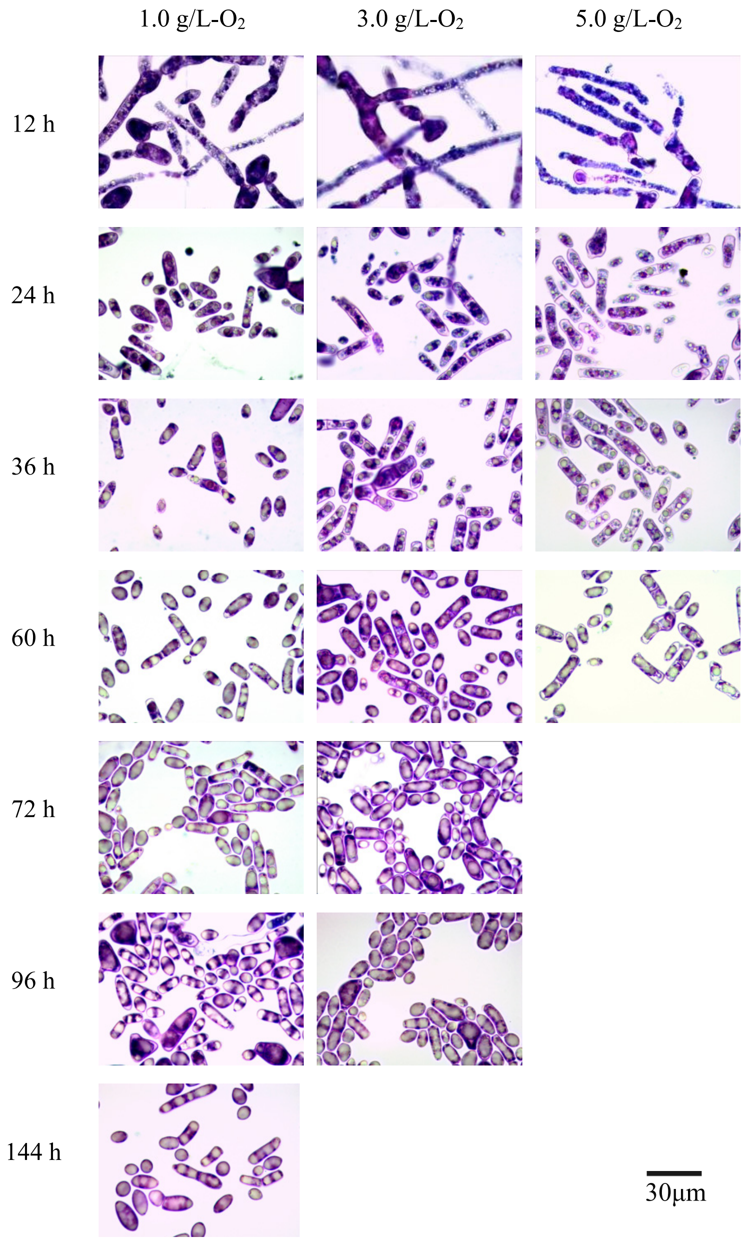

Figure S3

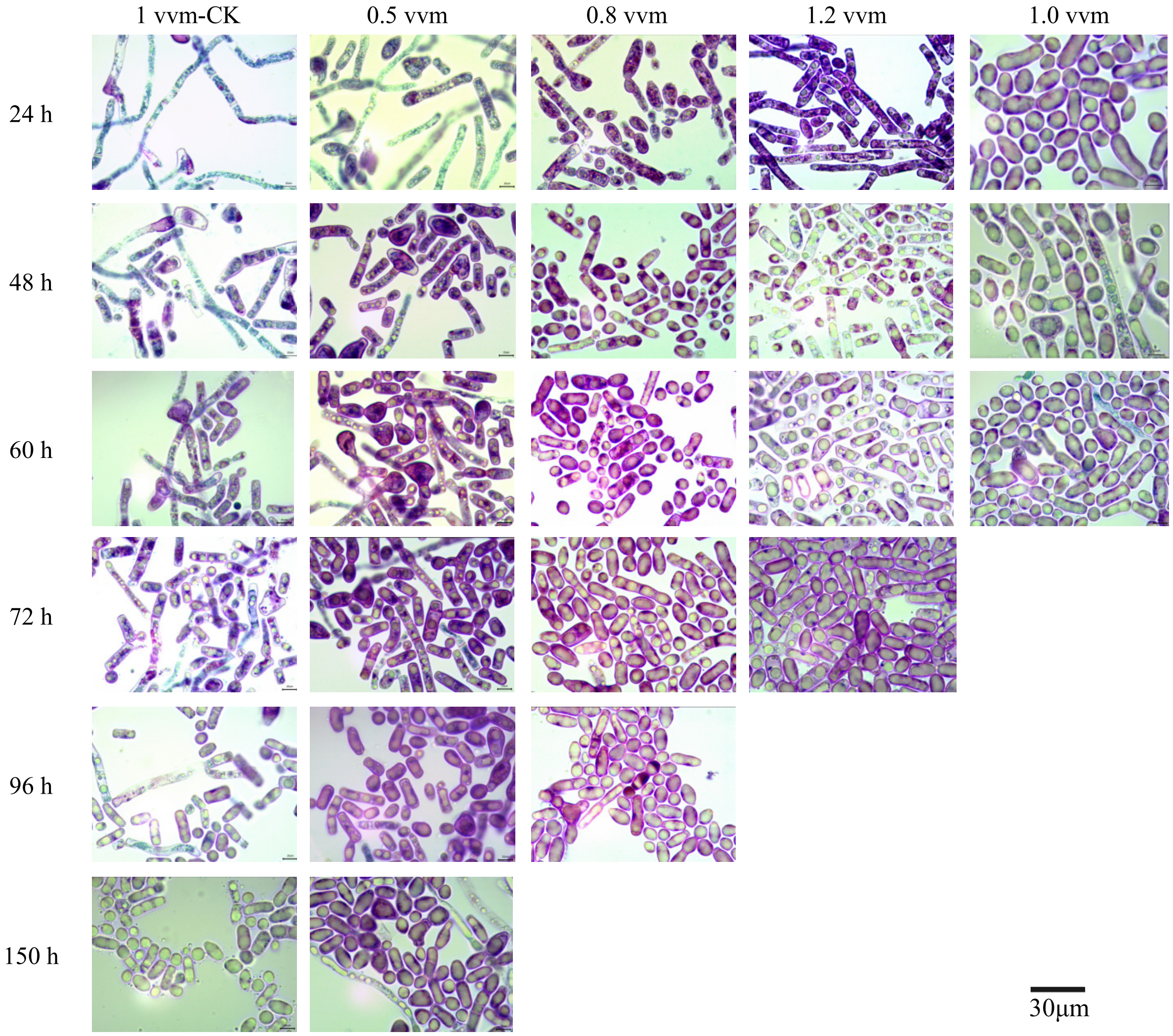

Figure S4

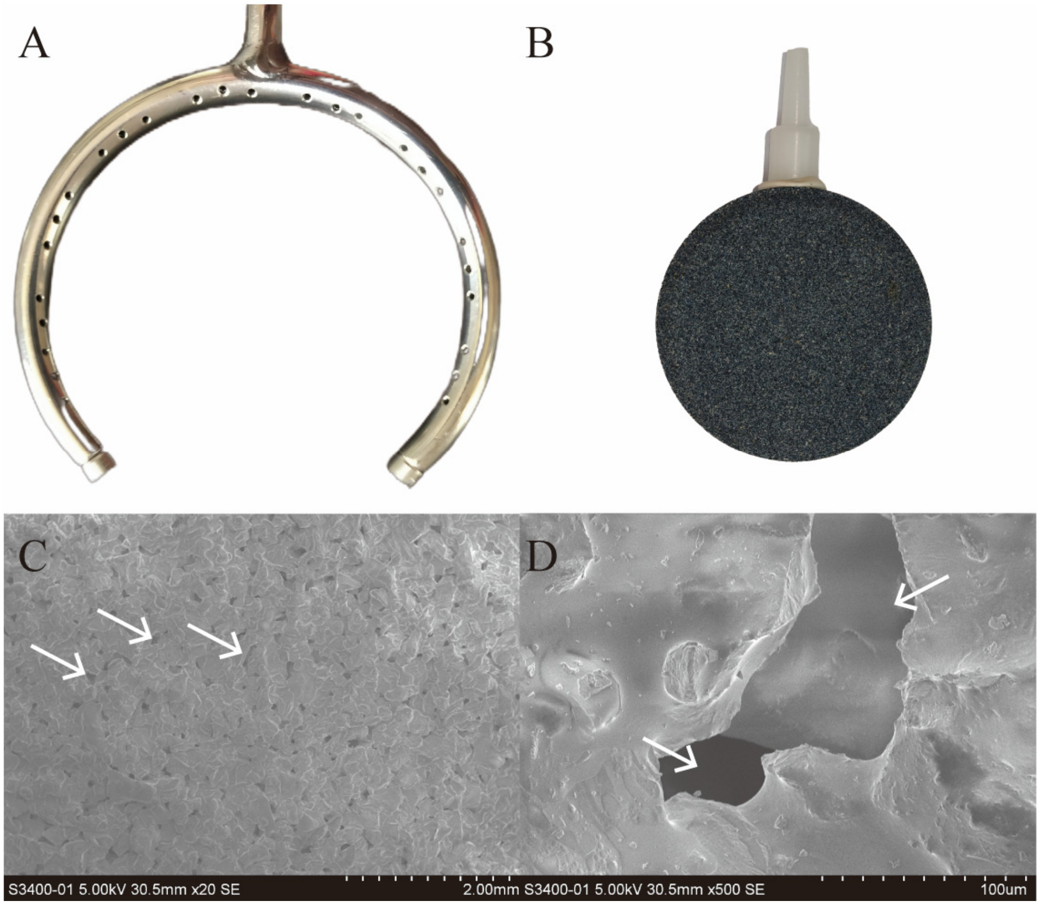
A
B
C
D
S3400-01 5.00kV 30.5mm x20 SE
2.00mm
S3400-01 5.00kV 30.5mm x500 SE
100um
